# Biodegradable Nanofibrillated Cellulose/Poly-(butylene adipate-co-terephthalate) Composite Film with Enhanced Barrier Properties for Food Packaging

**DOI:** 10.3390/molecules28062689

**Published:** 2023-03-16

**Authors:** Xiangyang Zhou, Guoqiang Yin, Yunchao Huang, Yuan Li, Dong Xie

**Affiliations:** 1 College of Chemistry and Chemical Engineering, Zhongkai University of Agriculture and Engineering, Guangzhou 510225, China; 2 Guangdong Biomaterials Engineering Technology Research Center, Institute of Biological and Medical Engineering, Guangdong Academy of Sciences, Guangzhou 510316, China; 3 Yingde Yunchao Polymer Material Co., Ltd., Qingyuan 510500, China

**Keywords:** nanofibrillated cellulose, green surfactant, biocomposites, packaging

## Abstract

Biodegradable composites consisting of Poly-(butylene adipate-co-terephthalate) (PBAT), thermoplastic starch, hydrophobically modified nanofibrillated cellulose (HMNC), and green surfactant (sucrose fatty acid ester) were prepared via the melt-mixing and film-blowing process (PBAT-HMNC). The composites were characterized using the Fourier transform infrared spectroscope (FT-IR), scanning electron microscope (SEM), and thermogravimetric analyzer (TGA). The mechanical and barrier properties were systematically studied. The results indicated that PBAT-HMNC composites exhibited excellent mechanical and barrier properties. The tensile strength reached the maximum value (over 13 MPa) when the HMNC content was 0.6% and the thermal decomposition temperature decreased by 1 to 2 °C. The lowest values of the water vapor transmission rate (WVTR) and the oxygen transmission rate (OTR) were obtained from the composite with 0.6 wt% HMNC, prepared via the film-bowing process with the values of 389 g/(m^2^·day) and 782 cc/(m^2^·day), which decreased by 51.3% and 42.1%, respectively. The *Agaricus* mushrooms still had a commodity value after 11 days of preservation using the film with 0.6 wt% HMNC. PBAT-HMNC composites have been proven to be promising nanocomposite materials for packaging.

## 1. Introduction

Environmentally friendly materials are research hotspots and a focus due to the “white pollution” issue. The excessive utilization of conventional plastic products, such as shopping bags, packaging films, bottles, courier bags, etc., has seriously threatened the environment and social development. Compared with metal, stone, and wood, plastic products have the advantages of low-cost and strong plasticity, which are widely used in many fields. However, the drawback of traditional plastic products is that they are non-biodegradable, resulting in a serious environmental issue. As reported, a plastic waste mountain was found in the ocean, and the area is increasing yearly [1,2]. Generally, plastic products such as shopping bags, packaging boxes, and agricultural mulch film are only used for a few minutes, but it takes hundreds of years or even longer for these to decompose in nature. Researchers focused on finding the ideal candidates to replace traditional plastic products. In this case, the development of green-based materials such as natural polymers (cellulose, chitosan, starch, alginate, etc.), man-made biodegradable polymers (Poly-(butylene adipate-co-terephthalate), poly (lactic acid), Polypropylene carbonate, Polyhydroxyalkanoates, Polybutylene succinate, etc.) are the focus of researchers in recent years, which will be conducive to the overall development of the economy and society, and the harmonious coexistence of human and nature [3,4,5].

Among those conventional packaging materials, plastic products are widely used due to the low cost and light-weight properties. In addition, such plastic products usually maintain functional properties, such as grease resistance and gas barrier properties. In recent years, green-based materials (cellulose and regenerated cellulose) or biodegradable plastics (PLA, PBAT, PHB, and PHBV) have become research hotspots in the field of packaging. Herein, Poly-(butylene adipate-co-terephthalate) (PBAT) is considered a completely biodegradable aliphatic aromatic polyester. PBAT is a biodegradable material with exceptional mechanical and film-forming properties. Despite exhibiting low water vapor resistance, PBAT displays excellent oxygen barrier properties, even more than that of low-density polyethylene (LDPE). Such qualities have earned PBAT polymers the reputation of being one of the more cost-effective alternatives to commodity petrochemical-based materials. The nanocomposite films were prepared with a different source of fillers and biodegradable polymers. The as-prepared composite films were systematically characterized to verify the effects of the different types of fillers on the morphological, water vapor barrier, thermal stability, color, optical, and antimicrobial properties. The results indicated that the biodegradable composite films showed improved properties (optical and mechanical). The composite films exhibited strong antibacterial activity [6]. PBAT-based biodegradable films incorporated with nano particles were prepared to improve the mechanical and water vapor barrier properties of PBAT film. The as-prepared nanocomposite films exhibited strong antibacterial activity against Escherichia coli and Listeria monocytogenes. The developed films have a high potential for being used as UV-screening and antimicrobial packaging films [7]. Other work also reported the development of antifungal bioplastic films based on poly-(lactic acid) (PLA) and poly-(butylene adipate-co-terephthalate) (PBAT) blends with incorporated trans-cinnamaldehyde using cast extrusion. The synthesized films reduced bacterial and fungal growth in bread, extending shelf-life for more than twenty days. Despite the advantages of PBAT, the relatively high-cost and low water vapor barrier property of PBAT in comparison to fossil-based polymers has impeded its extensive use in areas such as packaging. Therefore, the PBAT/PLA composite incorporated with the nano-polyhedral oligomeric silsesquioxane as a reactive compatibilizer was prepared using melt processing. The as-prepared biodegradable films exhibited improved water vapor, CO_2_ and O_2_ permeability, which offered a method for preparing high-performance biodegradable plastic packaging films [8]. In addition, nanocomposite technology has the potential to improve polymer properties and expand the applications of PBAT. Shankar and Rhim [9] prepared three different types of zinc oxide nanoparticles (ZnONP), and the PLA/PBAT nanocomposite films were then prepared with the reinforcement of ZnONP. The nanocomposites films showed improved optical and mechanical properties and exhibited strong antibacterial activity. The PBAT/silver nanoparticles (AgNPs) composite films were synthesized; the results indicated that the incorporation of AgNPs improved the mechanical and water vapor barrier properties. The developed films have a high potential for being used as UV-screening and antimicrobial packaging films [10]. In lieu of this, the effects of the fillers such as single-layered MMT nanosheets [11] and nanocellulose [12] on PBAT composites have also been examined. Nanofillers, such as MMT and nanocellulose, improve the barrier properties of the biodegradable substrate dramatically while being well-dispersed. In the reported work [11], the addition of the MMT, the biodegradable polymer, and the implemented orientation process resulted in a decrease in the viscosity of the composites and an increase in the crystallinity of PLA (up to 25%), and the wettability tests confirmed the synergic behavior of the selected polymer blend. However, the dispersion ability of the nanocellulose will dramatically affect the barrier property of the polymer film, this key factor has seldom improved in the above literatures.

Generally, the dispersion of the nanofillers in substrates is the key factor affecting the barrier property of the polymer matrices. Depending on the interaction between nanofillers and polymers, there are usually four different types of composite morphology that might be obtained: exfoliated; flocculated; intercalated; and aggregated. For the above types, the exfoliated one is the most ideal morphology to obtain dominant barrier properties for H_2_O, O_2_, CO_2_, etc., involving immense polymer penetration with the individual delaminated platelets and dispersed in the polymers randomly. Materials with excellent barrier properties are especially noticed by researchers. The use of renewable materials in packaging has earned many accolades [13]. Cellulose fiber is the most abundant biopolymer on earth and is utilized in numerous fields due to the advantages of being sustainable, biodegradable, and environmentally friendly. In recent years, many cellulose derivatives, including cellulose nanomaterials (cellulose nanocrystals (CNC), nanofibrillated cellulose (NFC) or cellulose nanofiber (CNF), and bacterial nanocellulose (BNC)), have been commercially produced with excellent properties for film-forming. These derivatives are relatively low-cost and have demonstrated excellent gas barrier properties. In addition, nanocellulose materials have great potential to improve the mechanical and barrier properties while being used as nanofillers in packaging film [14]. The activated hydroxyl groups are the principal structural components in cell walls, which can be easily modified with other functional groups via various derivation reactions including esterification, halogenation, oxidation, and etherification. Such modifications improve the properties of cellulose substantially, particularly the dispersibility. In this case, instead of using inorganic filler, hydrophobic-modified cellulose derivatives are usually used for enhancing the barrier property; hydrophobic-modified cellulose derivatives extend the water vapor transmission path, and thus do not permit the penetration of water through the molecular system [15,16]. However, most of the reported work carried out the chemical modification with many organic solvents, resulting in environmental pollution issues. Our previous work successfully prepared a novel composite consisting of dissolved cellulose fiber and microfibrillated cellulose for dye removal. The combination of microfibrillated cellulose with substrate significantly increased the adsorption capacity and mechanical properties of the biocomposite. The as-prepared cellulose-based composites were environmentally friendly and renewable bioadsorbents, which were not only applied in the field of environmental pollution control, but might also be promising for synthesizing the chemically modified cellulose derivatives products [17].

As a typical commercial biodegradable polymer, PBAT has the advantages of excellent processing performance and good barrier performance. However, the high-cost limits its application in the field of packaging. TPS is made using starch and plasticizer, which is biocompatible and low-cost. Filling TPS into PBAT will not only decrease the cost of the composite film, but also increase the biocompatibility of the final product. In this work, green-based biocomposites consisting of the biodegradable polymer (PBAT), thermoplastic starch, hydrophobically modified nanofibrillated cellulose (HMNC), and green surfactant (sucrose fatty acid ester) were prepared via the melt-mixing and film-blowing process without using any solvents, which has seldom been reported in other literature previously. A comparison of the properties of those biocomposites prepared using the solution-casting method was also systematically investigated. The morphology, thermal stability, and functional groups of the as-prepared biocomposites were systematically characterized. The mechanical properties and the influence factors by adding HMNC were systematically studied; the water vapor permeability (WVP) and oxygen transmittance rate (OTR) were also tested to provide a guide for maximizing the potential of the as-prepared biocomposite films for a variety of applications.

## 2. Results and Discussion

### 2.1. FT-IR Spectroscopy of PBAT-HMNC

**Figure 1:**
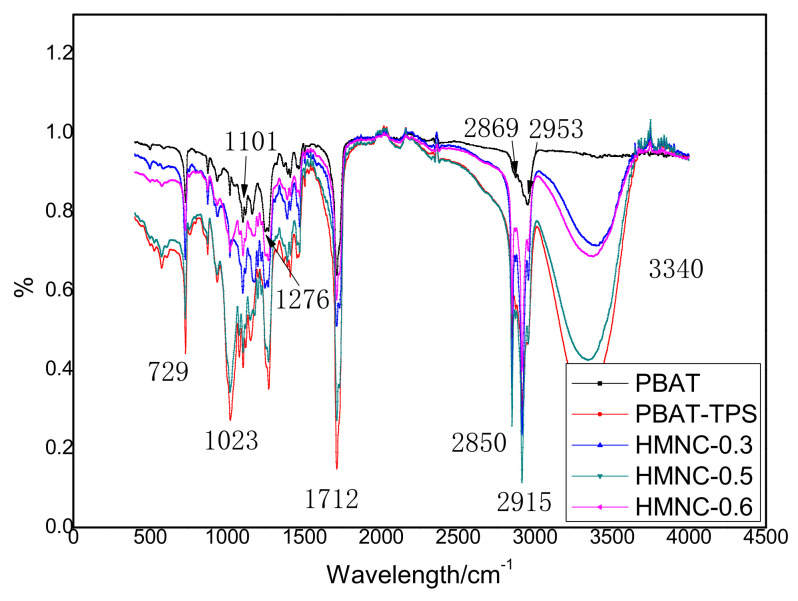
The FT-IR spectrum of PBAT-HMNC.

FT-IR was applied to verify the changes in the functional groups on PBAT, PBAT-TPS, and composites with different HMNC content (PBAT-HMNC-0.3, PBAT-HMNC-0.5, and PBAT-HMNC-0.6); the obtained results are indicated in Figure 1. The characteristic peaks of PBAT were identified (2953 cm^−1^, 2869 cm^−1^, 1712 cm^−1^, 1460–1450 cm^−1^, 1101 cm^−1^, 1276 cm^−1^, and 729 cm^−1^); [8] the peaks at 1101 cm^−1^ and 1276 cm^−1^ are attributed to the vibration adsorption of the C-O-C in the glucose ring from PBAT. The bending vibration absorption peak of the C-H on the para-disubstituted benzene ring is at 729 cm^−1^, and the absorption peak at 700–900 cm^−1^ further proves the existence of the benzene ring. The peaks at 1460–1450 cm^−1^ are moderate bending vibrations caused by -CH_2_, and the peaks at 2953 cm^−1^ and 2869 cm^−1^ are the stretching vibration absorption peaks of -CH_2_. The above peaks show the characteristic functional groups of PBAT [18]. In addition, the FTIR spectrum of all samples except PBAT gives the peaks at around 3400 cm^−1^ (the adsorption peak at 3340 cm^−1^), which might be due to the -OH group from the TPS and HMNC. The peak at 1712 cm^−1^ corresponds to the stretching vibration of -C=O group from PBAT and HMNC [19]. The stretching vibration absorption peaks of -CH_2_ (the peaks at 2953 cm^−1^ and 2869 cm^−1^) are deeper from the spectrum of PBAT-TPS, HMNC-0.3, HMNC-0.5, and HMNC-0.6, also corresponding to the addition of TPS and HMNC.

### 2.2. SEM Images of PBAT-HMNC

**Figure 2:**
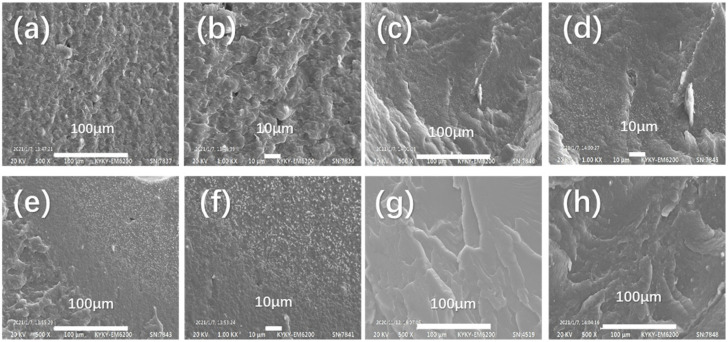
The SEM images of PBAT-HMNC composites: (**a**,**b**) PBAT-TPS composite; (**c**,**d**) HMNC-0.3; (**e**,**f**) HMNC-0.6; (**g**) PBAT; and (**h**) TPS.

The SEM can be applied to identify the morphology of the composite (PBAT-HMNC); the morphology of the composites (PBAT-TPS) and the composites with HMNC (PBAT-HMNC-0.3 and PBAT-HMNC-0.6) are compared to show whether the HMNC has been dispersed well in the substrate, and the obtained images are indicated in Figure 2. The compatibility between PBAT and TPS is excellent due to the smooth morphology obtained in Figure 2a,b. The TPS granules are dispersed well in the PBAT and the homogenous phase can be observed [20]. In addition, the HMNC also disperses ideally in the substrate (PBAT-TPS) via the application of extrusion and lab-scaled film blowing (from Figure 2c,d). As the content of HMNC increased, more HMNC fibers can be observed in the images (from Figure 2e,f). However, the ideal compatibility between HMNC and the substrate PBAT-TPS can also be observed; the fibers disperse well in the composites [21].

### 2.3. The Thermal Stability of the Composites (TG and DTG)

**Figure 3:**
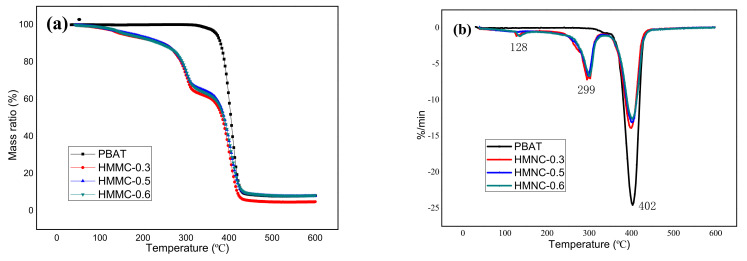
The DG and DTG curves of PBAT-HMNC: (**a**) the TG curves; (**b**) The DTG curves.

The material will undergo a sudden change in weight at a specific temperature, and TGA measurement can be used to determine the thermal degradation performance of the samples [22]. In this work, the thermal degradation performance of PBAT and PBAT-HMNC composites (PBAT-HMNC-0.3, PBAT-HMNC-0.5, and PBAT-HMNC-0.6) were tested using TGA and the corresponding results, including the TG and DTG curves, are indicated in Figure 3. Generally, PBAT shows a single thermal decomposition process at a certain temperature. From Figure 3, PBAT enters an obvious thermal degradation at 402 °C from Figure 3b (the thermal decomposition stage theoretically appears when the temperature is higher than 400 °C); during the process, some unstable components are decomposed into small molecule gases and condensable macromolecules. The residual of PBAT at 600 °C is less than 10%. In terms of the thermal decomposition stages of PBAT-HMNC composites, there are main three stages, which depend on the different contents of the matrix. Compared with the curves of TG and DTG, the first stage, which happens at 128 °C, is related to the evaporation of water from the composites (the temperature is higher than the boiling point of water); the mass ratio of the composites decreases no more than 10%. The second stage, obtained at 299 °C, corresponds to the thermal decomposition of TPS from the composites; the mass ratio of the composites decreases at around 30%, which matches the content of the TPS from the composite films. The third stage occurs at 402 °C with 54% mass loss of the composites, presumably due to the PBAT content. In a comparison of the DTG curves between PBAT, HMNC-0.3, HMNC-0.5, and HMNC-0.6, the thermal decomposition temperature of the third stage decreases by 1 or 2 °C, which might be due to the fillers (TPS and HMNC) changing the continuous structure of PBAT, thus destroying the continuous phase of PBAT and lowering thermal stability [23].

### 2.4. The Mechanical Properties of PBAT-HMNC Composites

**Figure 4:**
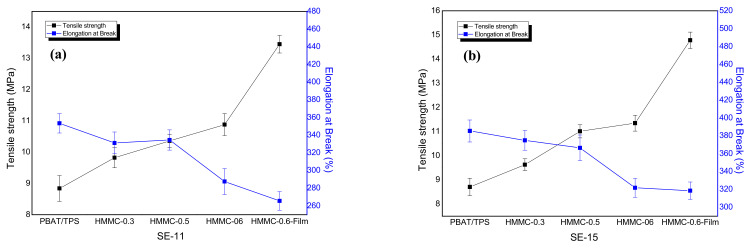
The tensile strength and elongation at break of PBAT-HMNC composites: (**a**) SE-11 and (**b**) SE-15.

Tensile strength can be measured using the tensile testing machine. In the tensile test, the tensile strength is shown by the maximum tensile stress of the sample until fracture. The elongation at break is expressed as a percentage (%) and usually refers to the ratio of the displacement of the sample at break to the original length. Tensile strength and elongation at break are extremely important factors for composite films; the corresponding results are revealed in Figure 4. The tensile strength and elongation at break exhibit the contrast trend with the addition of HMNC, the tensile strength increases and the elongation at break decreases with the increase in HMNC content. The HMNC is evenly dispersed in the PBAT matrix, and a well-dispersed structure can be observed (see the SEM images), resulting in increased tensile strength [24]. The elongation at break decreases with the increase in HMNC content, which might be due to the continuous phase of the PBAT molecule chains being destroyed, and defect points appear during the test. The sample breaks faster when the testing equipment pulls the samples.

In addition, the tensile strength reaches the maximum value when the HMNC content is 0.6% for the composite with both SE-11 and SE-15. The HMNC-0.6 film, produced by a film-blowing process, reveals the maximum tensile strength compared to the films with the HMNC contents of 0.3% and 0.5%. During the film-blowing process, the film forms with a vertical force, resulting in the formation of an oriented network where the HMNC and TPS reach an ideal dispersion state. The force from tensile testing equipment, therefore, will be transferred from the samples to HMNC and TPS while stretching, resulting in improved tensile strength of the composite films. This phenomenon is also demonstrated with the report that biaxial tensile processing can improve the film properties.

### 2.5. The Barrier Properties of PBAT-HMNC Composites

**Figure 5:**
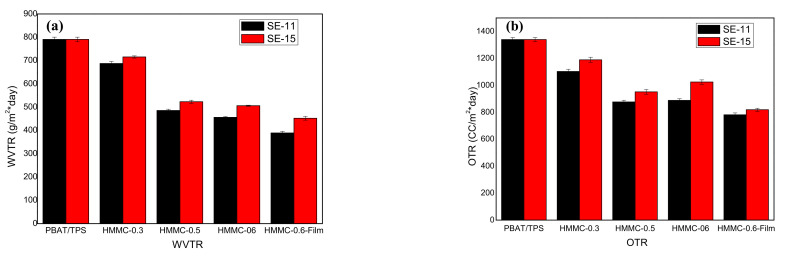
The WVTR (**a**) and OTR (**b**) of PBAT-HMMC composites (SE-11 and SE-15 added).

The barrier performance is an evaluation of the permeability of the packaging materials with respect to a certain penetration object. It is an important indicator for packaging materials, and also a basic function that the packaging materials must have, especially for food and medicine. The barrier properties of the composites have been tested and the corresponding results, including the WVTR and OTR, are indicated in Figure 5. The influence of the HMNC content on the PBAT/TPS film is also shown. As indicated, the PBAT/TPS composite has a relatively lower barrier property towards water vapor and oxygen, with the WVTR and OTR of around 800 g/(m^2^·day) and 1350 cc/(m^2^·day), respectively. The WVTR and OTR decrease with the increase in HMNC content. The lowest values of WVTR and OTR are 389 g/(m^2^·day) and 782 cc/(m^2^·day) for PBAT/TPS film with 0.6 wt% HMNC produced via a film-blowing process, which is 51.3% and 42.1% lower than that of the PBAT/TPS composite without HMNC. Generally, the HMNC can improve the gas barrier property, thus reducing gas dissolution on the surface of the composite film and reducing the gas diffusion rate. The improved gas barrier property is mainly due to the “multi-path” effect; in addition, the molecular chain of the HMNC is flexible and easy to wind, which forms a dense three-dimensional network. Meanwhile, the film-blowing process makes the HMNC easier to disperse evenly and form an oriented structure, resulting in a higher gas barrier property of the composite film [25].

### 2.6. The Freshness Preservation Evaluation

**Figure 6:**
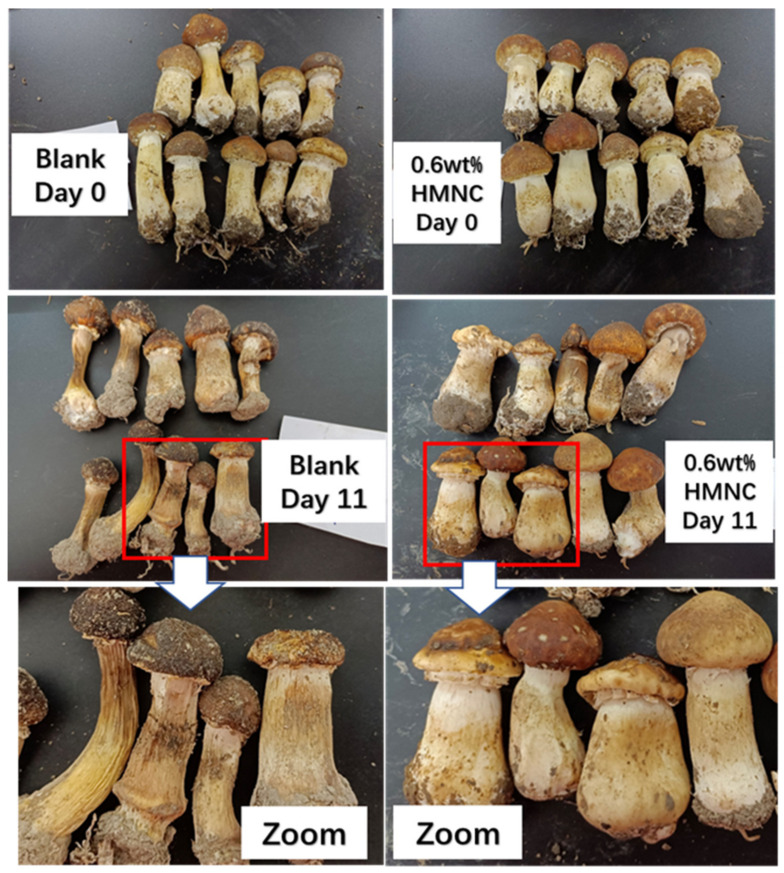
The freshness preservation evaluation of *Agaricus* mushrooms using commercial PE film and PBAT-HMNC (PBAT-HMNC-0.6).

As a fresh-keeping packaging material, better barrier performance is a critical parameter. The research shows that film with a certain barrier performance and certain permeability has the best fresh-keeping effect for fruits and vegetables. The freshness preservation evaluation of the high-valued mushroom (*Agaricus* mushroom) was carried out using PBAT/TPS film with 0.6 wt% HMNC and commercial polyethylene punch film as the packing films at the conditions of 5 °C and normal humidity. The weightlessness rate was calculated and the morphology was recorded by a digital camera to verify the changes in the quality of the products; the obtained morphology results are indicated in Figure 6. The *Agaricus* mushrooms still had a commodity value after 11 days of preservation using the film prepared in this work, and the weightlessness rate was 18.51%. In addition, the mushrooms packed in the PE film lost 49.5% of the total weight after 11 days of preservation. Most of the mushrooms were dry and lost their commercial value. The reason might be due to the fact that fungi, fruits, and vegetables also breathe in the packaging films and this requires a certain amount of oxygen in the film (the film needs a certain oxygen permeability). In addition, the water vapor generated by respiration also needs to be removed in time, which can easily lead to the deterioration of the mushrooms, fruits, and vegetables. In a reported work, the CNC has been successfully filled in biodegradable film and applied to the fresh-keeping of mango, which proved that biodegradable composite films were ideal candidates for food packaging [26]. In this work, all the raw materials are food contact materials and the as-prepared packaging films can also be used to extend the freshness period of common fruits and vegetables. The PBAT/TPS biodegradable film was proved to be the most proper candidate to pack edible fungi such as *Agaricus* mushrooms, oyster mushrooms, matsutake mushrooms, etc.

## 3. Materials and Methods

### 3.1. Materials

Poly-(butylene adipate-co-terephthalate) (PBAT, C1200, density 1.25–1.27 g/cm^3^, melt volume flow (MVR) (190 °C/2.16 kg) 2.5–4.5 cm^3^/10 min) was purchased from BASF, Ludwigshafen, Germany; nanofibrillated cellulose (NFC, solid content 4.5% ± 0.5%) was obtained from Guilin Qihong Technology Co., Ltd. (Guilin, China). Corn starch, glycerol, palm wax, sucrose fatty acid ester (SE-11 and SE-15, hydrophile lipophile balance, hydrophilic and lipophilic equilibrium values (HLB values) are 11 and 15, ethylene, and other reagents were used as received from Macklin reagent Co. (Shanghai, China)

### 3.2. Preparation of the Palm Wax Emulsion

First, 100 g palm wax was added to a beaker and heated up to about 85 °C, the wax was melted and then a total volume of 400 mL surfactant (SE-11 and SE-15) (1 wt% based on the weight of palm wax) aqueous solution with a temperature of 90 °C was added to the melted palm wax. After 3 min, emulsification took place using the high-speed homogenizer (BRS-200, Anhui Bunkin Chemical Machinery Co., Ltd., Hefei, China); the palm wax emulsion was obtained and quickly transferred to the ice water bath. Finally, the emulsion was obtained after being cooled down to room temperature and the resulting emulsion was labeled as PWE (white color).

### 3.3. Preparation of the Hydrophobically Modified Nanofibrillated Cellulose (HMNC)

The HMNC was prepared via an emulsion modification with the addition of PWE. First, the solution with 5 wt% of the nanofibrillated cellulose (NFC) was prepared in three-necked flasks, the PWE was then added with a fixed weight ratio (5% towards the content of NFC). The matrix solution was then placed on a stirrer after being fully dispersed. The chains of the cellulose were loaded with the PWE; the hydrophilicity of NFC was changed by the reaction that occurred between -OH on cellulose chains and PWE for a one-hour modification process. The water was then removed via filtration and the modified NFC was then obtained (HMNC) [27]. The resulting HMNC was then dried at 80 °C in a vacuum oven for 24 h, ground and stored in a dry cabinet for future utilization.

### 3.4. Preparation of PBAT- HMNC Composite Films

The PBAT-HMNC with four different HMNC concentrations (0, 0.3, 0.5, and 0.6 wt%), PBAT, and TPS were prepared using a melt extrusion in a twin-screw extruder (H1GD 20, Guangzhou Hartek Technologies Co., Ltd., Guangzhou, China 150 °C, 100 rpm, and residence time of 5 min; the TPS was prepared by mixing the 74 wt% of corn starch with the 26 wt% of glycerol under the conditions of 140 °C, 100 rpm, and residence time of 5 min). The PBAT was dried for at least 1 h at 80 °C before use. The masterbatch of PBAT-HMNC (marked as PBAT-HMNC-0.3, PBAT-HMNC-0.5, PBAT-HMNC-0.6) was obtained from the cutter, and the composite films were produced via lab-scaled film-blowing equipment (XH-432-25, Dongguan Xihua Testing Machine Co., Ltd., Dongguan, China). The films were stored properly for further testing and application. Subsequently, the masterbatch of PBAT-HMNC was hot pressed (XH-406BWP-30-300, Dongguan Xihua Testing Machine Co., Ltd., Dongguan, China). Plates with dimensions of 25 mm × 1 mm were obtained for tensile testing.

Meanwhile, the non-solvent process was carried out by adding the materials (PBAT, TPS, NFC, surfactants, and wax) directly into the twin-screw extruder, followed by the film-blowing and hot-pressing processes for preparing the corresponding films and plates for further characterization.

### 3.5. Characterization

The surface morphology of PBAT-HMNC was tested via a Scanning Electron Microscope (SEM, FEI Phenom Prox, 5 kV, Phenom-world B.V., Eindhoven, The Netherlands). Samples were prepared by cutting before being sprayed with a thin gold layer. The functional groups of the synthesized samples were measured using FTIR spectra (Invenio S, Bruker Corporation, Billerica, MA, USA) and the samples were tested directly with an ATR spectrophotometer. The data were collected 32 times at a resolution of 1 cm^−1^. The thermal stability of the composite films was examined using thermogravimetric analysis (TG 209 F3 Tarsus, Tarsus GmbH, Berlin, Germany); the test was carried out from 35 °C to 700 °C at a heating rate of 20 °C/min under nitrogen condition (flow rate of 20 cm^3^/min). The tensile tests were performed in a Testing Machine (Model E43, MTS Industrial System (China) Co., Ltd., Shanghai, China) according to GB/T 1040.3 Standard. The dog bone specimens were prepared before testing. Five specimens were analyzed for each composition. The tensile strength and elongation at break were obtained from the tests.

### 3.6. The Measurement of the Oxygen and Water Vapor Barrier Properties

The gas barrier properties (OTR and WVTR) were characterized using Oxygen Permeation Analyzer (OX-TRAN, Ametek Mocon, Minneapolis, MN, USA) and Water Vapor Permeability (Permatran-W 1/50G, Ametek Mocon, Minneapolis, MN, USA). During the WVTR test, the sample is installed in the cavity, and a certain humidity difference is formed on both sides of the sample. The water vapor will penetrate through the sample from the high-humidity side to the low-humidity side under the action of the humidity difference. Through the measurement of the water vapor penetration, the WVTR of the samples can be obtained. The OTR test is carried out with similar mechanism via Oxygen Permeability (OP) Analyzer.

## 4. Conclusions

Biodegradable composites consisting of HMMC and PBAT/TPS were successfully prepared via the extrusion and film-blowing processes, followed by the systematic characterization of the SEM, FTIR, mechanical, and barrier properties. The resulting PBAT-HMMC composites exhibited excellent mechanical and barrier properties. The lowest values of WVTR and OTR were obtained from the composite with 0.6 wt% HMMC, prepared via the film-blowing process. Overall, PBAT-HMMC composites have been proven to be easy to prepare, environmentally friendly, and promising gas barrier nanocomposite materials for packaging, showing a great economic value and the application can be readily extended to other fields, such as agricultural mulch, medicine capsules, etc.

## Data Availability

The data presented in this study are available in the article. The data sets generated during and/or analyzed during the current study are available from the corresponding author upon reasonable request.

## References

[1] Dupont H., Laurichesse E., Héroguez V., Schmitt V. (2021). Green Hydrophilic Capsules from Cellulose Nanocrystal-Stabilized Pickering Emulsion Polymerization: Morphology Control and Spongelike Behavior. Biomacromolecules.

[2] Li Y., Chen M.D., Wan X., Zhang L., Wang X., Xiao H. (2017). Solvent-free synthesis of the cellulose-based hybrid beads for adsorption of lead ions in aqueous solutions. RSC Adv..

[3] Lu P., Zhao H., Zheng L., Duan Y., Wu M., Yu X., Yang Y. (2022). Nanocellulose/Nisin Hydrogel Microparticles as Sustained Antimicrobial Coatings for Paper Packaging. ACS Appl. Polym. Mater..

[4] Huang X., Wang Y., Wang Y., Yang L. (2023). A Facile One-Pot Preparation and Properties of Nanocellulose-Reinforced Ionic Conductive Hydrogels. Molecules.

[5] Poulose A., Parameswaranpillai J., George J.J., Gopi J.A., Krishnasamy S., Dominic C.D.M., Hameed N., Salim N.V., Radoor S., Sienkiewicz N. (2022). Nanocellulose: A Fundamental Material for Science and Technology Applications. Molecules.

[6] Ahankari S.S., Subhedar A.R., Bhadauria S.S., Dufresne A. (2021). Nanocellulose in food packaging: A review. Carbohyd. Polym..

[7] Shankar S., Rhim J.W. (2016). Tocopherol-mediated synthesis of silver nanoparticles and preparation of antimicrobial PBAT/silver nanoparticles composite films. LWT-Food Sci. Technol..

[8] Ren P.G., Liu X.H., Ren F., Zhong G.J., Ji X., Xu L. (2017). Biodegradable graphene oxide nanosheets/poly-(butylene adipate-co-terephthalate) nanocomposite film with enhanced gas and water vapor barrier properties. Polym. Test..

[9] Shankar S., Rhim J.W. (2019). Effect of types of zinc oxide nanoparticles on structural, mechanical and antibacterial properties of poly(lactide)/poly (butylene adipate-co-terephthalate) composite films. Food Packag. Shelf Life.

[10] Muthuraj R., Misra M., Mohanty A.K. (2018). Biodegradable compatibilized polymer blends for packaging applications: A literature review. J. Appl. Polym. Sci..

[11] Ludwiczak J., Frąckowiak S., Leluk K. (2021). Study of Thermal, Mechanical and Barrier Properties of Biodegradable PLA/PBAT Films with Highly Oriented MMT. Materials.

[12] Li Y., Xiao H., Pan Y., Wang L. (2018). Novel Composite Adsorbent Consisting of Dissolved Cellulose Fiber/Microfibrillated Cellulose for Dye Removal from Aqueous Solution. ACS Sustain. Chem. Eng..

[13] Saremi R., Borodinov N., Laradji A.M., Sharma S., Luzinov I., Minko S. (2020). Adhesion and Stability of Nanocellulose Coatings on Flat Polymer Films and Textiles. Molecules.

[14] Perumal A.B., Nambiar R.B., Moses J.A., Anandharamakrishnan C. (2022). Nanocellulose: Recent trends and applications in the food industry. Food Hydrocoll..

[15] Oyeoka H.C., Ewulonu C.M., Nwuzor I.C., Obele C.M., Nwabanne J.T. (2021). Packaging and degradability properties of polyvinyl alcohol/gelatin nanocomposite films filled water hyacinth cellulose nanocrystals. J. Bioresour. Bioprod..

[16] Wang W., Gu F., Deng Z., Zhu Y., Zhu J., Guo T., Song J., Xiao H. (2021). Multilayer surface construction for enhancing barrier properties of cellulose-based packaging. Carbohyd. Polym..

[17] Li Y., Xie D., Xiao J., Wu W., Zhang L., Xiao H., Chen J. (2020). Dual responsive copolymers-grafted microfibrillated cellulose composites for removing lead ions from aqueous solution. J. Clean. Prod..

[18] Moustafa H., Guizani C., Dupont C., Martin V., Jeguirim M., Dufresne A. (2017). Utilization of Torrefied Coffee Grounds as Reinforcing Agent to Produce High-Quality Biodegradable PBAT Composites for Food Packaging Applications. ACS Sustain. Chem. Eng..

[19] Xiong S.J., Pang B., Zhou S.J., Li M.K., Yang S., Wang Y.Y., Shi Q., Wang S.F., Yuan T.Q., Sun R.C. (2020). Economically Competitive Biodegradable PBAT/Lignin Composites: Effect of Lignin Methylation and Compatibilizer. ACS Sustain. Chem. Eng..

[20] Wei X.Y., Ren L., Sun Y.N., Zhang X.Y., Guan X.F., Zhang M.Y., Zhang H.X. (2021). Sustainable composites from biodegradable poly(butylene succinate) modified with thermoplastic starch and poly(butylene adipate-co-terephthalate): Preparation and performance. New J. Chem..

[21] Perumal A.B., Nambiar R.B., Sellamuthu P.S., Sadiku E.R., Li X., He Y. (2022). Extraction of cellulose nanocrystals from areca waste and its application in eco-friendly biocomposite film. Chemosphere.

[22] Perumal A.B., Sellamuthu P.S., Nambiar R.B., Sadiku E.R. (2018). Development of polyvinyl alcohol/chitosan bio-nanocomposite films reinforced with cellulose nanocrystals isolated from rice straw. Appl. Surf. Sci..

[23] Paul U.C., Fragouli D., Bayer I.S., Zych A., Athanassiou A. (2021). Effect of Green Plasticizer on the Performance of Microcrystalline Cellulose/Polylactic Acid Biocomposites. ACS Appl. Polym. Mater..

[24] Gregorich N., Kanhere S., Stutts J., Bethel K., Tindall G., Lynn B., Ogale A.A., Thies M.C., Davis E.M. (2023). Enhanced Mechanical Properties of Composite Hydrogels Containing Fractionated and Purified Lignin. ACS Appl. Polym. Mater..

[25] Santos J.F., Valls C., Cusola O., Roncero M.B. (2021). Improving Filmogenic and Barrier Properties of Nanocellulose Films by Addition of Biodegradable Plasticizers. ACS Sustain. Chem. Eng..

[26] Perumal A.B., Sellamuthu P.S., Nambiar R.B., Sadiku E.R., Phiri G., Jayaramudu J. (2018). Effects of multiscale rice straw (*Oryza sativa*) as reinforcing filler in montmorillonite-polyvinyl alcohol biocomposite packaging film for enhancing the storability of postharvest mango fruit (*Mangifera indica* L.). Appl. Clay Sci..

[27] Paula G.S., Lídia E.M., Lucia F., Izabel D.J., Silvia G. (2016). Influence of incorporation of starch nanoparticles in PBAT/TPS composite films. Polym. Int..

